# Stiff-Soft Hybrid Biomimetic Nano-Emulsion for Targeted Liver Delivery and Treatment of Early Nonalcoholic Fatty Liver Disease

**DOI:** 10.3390/pharmaceutics16101303

**Published:** 2024-10-07

**Authors:** Juan Li, Mingxing Yin, Maoxian Tian, Jianguo Fang, Hanlin Xu

**Affiliations:** 1 School of Pharmacy, Hubei University of Chinese Medicine, Wuhan 430065, China; 2 Department of Pharmacy, Tongji Hospital, Tongji Medical College, Huazhong University of Science and Technology, Wuhan 430030, China

**Keywords:** drug delivery system, biomimetic nano-emulsion, dihydromyricetin, liver targeting, nonalcoholic fatty liver disease

## Abstract

**Background:** Nonalcoholic fatty liver disease (NAFLD) poses a risk for numerous metabolic diseases. To date, the U.S. Food and Drug Administration has not yet approved any medications for the treatment of NAFLD, for which developing therapeutic drugs is urgent. Dihydromyricetin (DMY), the most abundant flavonoid in vine tea, has been shown to be hepatoprotective. Its application was limited by low bioavailability in vivo; **Methods:** In order to improve the bioavailability of DMY and achieve liver-targeted delivery, we designed a DMY-loaded stiff-soft hybrid biomimetic nano drug delivery system (DMY-hNE). The in vivo absorption, distribution, pharmacokinetic profiles, and anti-NAFLD efficacy of DMY-hNE were studied; **Results:** DMY-hNE was composed of a stiff core and soft shell, which led to enhanced uptake by gastrointestinal epithelial cells and increased penetration of the mucus barrier, thus improving the in vivo absorption, plasma DMY concentration, and liver distribution versus free DMY. In an early NAFLD mouse model, DMY-hNE effectively ameliorated fatty lesions accompanied with reduced lipid levels and liver tissue inflammation; **Conclusions:** These findings suggested that DMY-hNE is a promising platform for liver drug delivery and treatment of hepatopathy.

## 1. Introduction

Nonalcoholic fatty liver disease (NAFLD) is a spectrum of liver diseases related to insulin resistance and metabolic disorders, including fatty liver, steatohepatitis, cirrhosis, and hepatocellular carcinoma [1,2,3]. Recently, changes in lifestyle have led to a gradual increase in obesity rates, which has added to the burden of NAFLD, contributing to a global prevalence of NAFLD of approximately 25% [3,4,5,6]. NAFLD is a hazardous indicator for many metabolic diseases, including obesity, diabetes mellitus, hyperlipidemia, and hypertension [7]. According to the American Association for the Study of Liver Diseases’ practice guidelines, patients with NAFLD should make lifestyle changes and treat the metabolic syndrome linked to the liver disease, while those with biopsy-proven nonalcoholic steatohepatitis (NASH) and fibrosis should receive pharmacological treatments [8]. Although glucose-lowering drugs (e.g., metformin, pioglitazone, glucagon-like peptide-1 (GLP-1) analogues), antioxidants (e.g., vitamin E), lipid-lowering drugs (e.g., statins), and hepatoprotective drugs (e.g., obeticholic acid, and ursodeoxycholic acid) are currently used for the treatment of NAFLD [9,10,11,12], there are a variety of adverse effects associated with these medications such as an increase in mortality at high doses of vitamin E, risk of increased body weight and fluid retention with pioglitazone, and gastrointestinal adverse effects with GLP-1 analogues. In addition, to date, neither the U.S. Food and Drug Administration nor the European Medicines Agency has approved a drug for the treatment of NAFLD [10]. Therefore, the search for effective drug candidates to improve NAFLD remains urgent.

Dihydromyricetin (DMY), also known as ampeloptin, is a flavonoid extracted from the stems and leaves of vine tea [13]. As the pharmacological substance of vine tea, DMY is widely used in the treatment of liver injury, fatty liver disease, and acute liver failure [14] due to the pharmacological effects, such as anti-inflammatory, antioxidant, hypoglycaemic, hypolipidemic, hepatoprotective, and cardiovascular protection [15]. It is reported as a potential molecule for the treatment of NAFLD. DMY exerts therapeutic effects on NAFLD through multiple hepatoprotective mechanisms, including regulating lipid metabolism/insulin resistance and exerting sirtuins-dependent/anti-inflammatory effects [16]. Nevertheless, DMY has limitations such as slight solubility in cold water (0.2 mg/mL at 25 °C [17]), poor chemical stability, low bioavailability (~4%) [18], and resulting insufficient liver distribution [19].

To overcome the limitations of DMY, varieties of DMY-loaded nano-formulations were developed, such as liposomes [20], polymer micelles [21], nanoparticles (NP) [22,23], phospholipid complex [24], inclusion complex, and solid dispersion [25]. Challengingly, before getting into blood circulation, an oral drug delivery system must pass through the harsh physiological environment and biological barriers of the gastrointestinal tract (GI), such as the gastrointestinal mucus layer and tightly connected epithelial cells [26,27,28]. Recent studies have illustrated that the rigidity of nanoparticles plays a significant role in regulating their transport in the GI [29]. In the soft-NP, semi-NP, and stiff-NP, the soft-NP has the ability to penetrate the gastrointestinal mucus layer and the stiff-NP can be taken up by gastrointestinal epithelial cells, while the semi-NP has the best mucus layer penetration and epithelial cell uptake [29,30]. In addition, dietary lipids such as triglycerides (TG) are absorbed and processed by epithelial cells in the GI and assembled with associated proteins to form chylomicrons (CM), which can be transported throughout the body via the intestinal lymphatics [31,32]. Co-administration of drugs with lipid preparations can increase intestinal lymphatic absorption. In recent years, researchers have found that the use of biomimetic nanocarriers to mimic key components of the lipid absorption pathway (e.g., TG and CM structures) can be used for effective transport into the intestinal lymphatic system [32].

**Figure 1:**
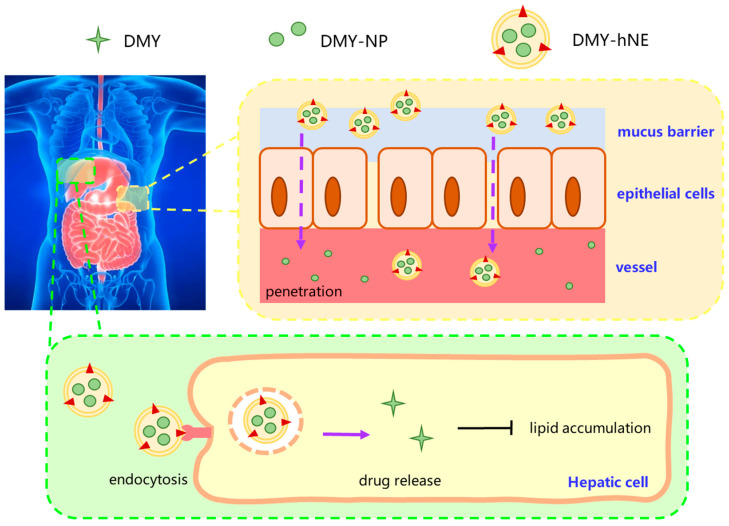
Schematic illustration of in vivo absorption and liver-targeted delivery of DMY-loaded stiff-soft hybrid nano-emulsion (DMY-hNE).

Based on the above background, this study designed a DMY-loaded stiff-soft hybrid biomimetic nano-emulsion (DMY-hNE) for targeted liver delivery and treatment of NAFLD (Figure 1). DMY was encapsulated in poly(lactic acid) (PLA) to form a stiff nanoparticle core (DMY-NP) which promotes drug penetration. Hybrid biomimetic nano-emulsion (DMY-NE) performed as the soft shell which was beneficial for penetrating the mucus layer of the GI. The soft shell of DMY-hNE mimicked the CM in the absorption process of fat, which is emulsified by bile. DMY-NE with a biomimetic soft shell could pass through the mucus layer, and DMY-NP was subcutaneously taken up by gastrointestinal epithelial cells and successfully entered into blood circulation. CM-like particles could accumulate in liver, realizing passive liver-targeted drug delivery. The stiff-soft hybrid biomimetic nano-emulsion (hNE) may be a promising carrier for oral liver-targeted therapy of hepatopathy.

## 2. Materials and Methods

### 2.1. Materials

(+)-DMY was prepared by our laboratory with purity above 99% [33]. PLA (Mw: 18,000–24,000), soy lecithin, cholesterol, and coumarin-6 (C6) were purchased from Sigma-Aldrich (Saint Louis, MO, USA). Glycerol monooleate was obtained from Macklin (Shanghai, China). The lipophilic fluorescent dye (DiR) was from Dalian Meilun Biotechnology Co. Ltd. (Dalian, China). Metformin hydrochloride (MET) was bought from Wuhan Kerui Biotechnology Co. Ltd. (Wuhan, China). Triglyceride (TG), total cholesterol (TC), low-density lipoprotein (LDL), high-density lipoprotein (HDL), alanine transaminase (ALT), aspartate transaminase (AST), alkaline phosphatase (ALP), urea nitrogen (BUN), malondialdehyde (MDA), γ-glutamyl transpeptidase (GGT), nitric oxide (NO), reduced glutathione (GSH), superoxide dismutase (SOD), and hydrogen peroxide (H_2_O_2_) assay kits were all purchased from Nanjing Jiancheng Institute (Nanjing, China). Organic chemicals like methanol, acetone, dichloromethane, and formic acid were of analytical grade and produced from Sinopharm (Shanghai, China).

Male C57BL/6J mice and Sprague-Dawley (SD) rats were purchased from Beijing Vital River Laboratory Animal Technology Co., Ltd. (Wuhan Brand, China). All the animals were maintained under specific pathogen-free (SPF) conditions in the Animal Centre of Huazhong University of Science and Technology, Wuhan, China. All animals were treated according to the regulations of Chinese law and the study was approved by the Institutional Animal Care and Use Committee of Huazhong University of Science and Technology ((2023) IACUC Number: 3989). Before the experiments, all animals were fasted overnight with free access to water.

### 2.2. Preparation and Characterization of DMY-hNE

DMY-hNE was prepared in two steps. Briefly, 40 mg of (+)-DMY and 10 mg of PLA were dissolved in 0.3 mL of acetone, after which the mixture was dropped into 6 mL of dichloromethane solution containing 10 mg of soybean lecithin, 5 mg of cholesterol, and 120 mg of glyceryl monooleate under constant stirring to prepare oil-phase DMY-NP. The second step was to drop the oil-phase DMY-NP into deionized water at a volume ratio of 1:5. Then, the mixture was emulsificated using an ultra-sonicator (Scientz 950E, Ningbo Scientz Biotechnology Co. Ltd., Ningbo, China) at 250 W for 2 min and the organic solvent was evaporated to obtain DMY-hNE. C6-loaded stiff-soft hybrid biomimetic nano-emulsion (C6-hNE) and DiR labeled stiff-soft hybrid biomimetic nano-emulsion (DiR-hNE) were prepared using C6 and DiR instead of DMY, respectively.

DMY-hNE was assessed for particle size, zeta potential, morphology, and encapsulation efficiency. The particle size and zeta potential of DMY-hNE were determined by dynamic light scattering (DLS, Zetaplus, Brookhaven Instruments Co., Nashua, NH, USA) after DMY-hNE was diluted by deionized water to a transmittance index of ~100 kcps. The morphology of DMY-hNE was observed through a transmission electron microscope (TEM, JEM-1230, Jeol Ltd., Tokyo, Japan). The encapsulation efficiency (EE%) was studied using the ultrafiltration method. Briefly, nano-emulsion was introduced into a centrifugal filter tube and centrifuged. The phase solution under centrifugation was collected and diluted with methanol. After that, the amount of free DMY was detected by high-performance liquid chromatography (HPLC, Waters e2695, Waters Co., Taunton, NH, USA). EE% was calculated using Equation (1) [34]:

EE(%) = (Wtotal − Wfree) ÷ Wtotal × 100%(1)

### 2.3. Stability

To estimate the stability of DMY-hNE, DMY-hNE samples were dispersed in phosphate buffered saline (PBS), fetal bovine serum (FBS), and water, which were stored at 4 °C. At the 1st, 2nd, 3rd, 4th, and 5th day, the particle size of the diluted samples was measured by DLS.

### 2.4. In Vitro Drug Release

In vitro drug release of DMY-hNE was detected using a dialysis method. Briefly, 1 mL of DMY suspension or DMY-hNE was added into dialysis bags (MWCO: 3500 Da). To mimic the situation of DMY-hNE in the GI, the dialysis bags were immersed in 50 mL of pH 1.2 and pH 6.8 standard release media in US Pharmacopeia (USP), with constant shaking at 37 °C. At determined time intervals (0.083, 0.25, 0.5, 0.75, 1, 2, 3, 4, 6, 8, 12 h), 1 mL of the release media was collected and replaced with an equal volume of fresh media. The released DMY was measured by HPLC, and the cumulative DMY release profiles of DMY-hNE and free DMY in pH 1.2 and pH 6.8 were plotted, respectively.

### 2.5. In Vivo Drug Uptake

C57BL/6J mice (male, 8 w) were randomly divided into three groups (n = 3 for each group: Free C6, C6+vehicle, and C6-hNE) and fasted overnight with free access to water. Free C6, C6+vehicle, and C6-hNE were intragastrically (i.g.) administered to the mice. At determined time intervals (0.5, 1, 2 h), the mice were sacrificed, and the stomach and small intestinal were collected, frozen, and prepared for fluorescence observation.

### 2.6. Ex Vivo Imaging

To study the liver-targeted delivery of hNE, DiR labeled nano-emulsions were used as probes. C57BL/6J mice (male, 8 w) were divided into two groups at random (n = 12 for each group: Free DiR and DiR-hNE) and then fasted overnight with access to water. Following i.g. administration of free DiR and DiR-hNE, the mice in each group were sacrificed at 1, 4, 8, and 24 h, respectively. The heart, liver, spleen, lung, and kidney were collected and analyzed using a living imaging system (Pearl Trilogy, LI-COR Co., Lincoln, NE, USA).

### 2.7. Pharmacokinetics

Healthy SD rats (male, body weight ~250 g) were randomly divided into free DMY and DMY-hNE groups (n = 3). After an overnight fast, rats in the two groups were i.g. administrated with free DMY suspension and DMY-hNE at an equivalent dose of 25 mg DMY/kg, respectively. At desired time intervals (0.083, 0.25, 0.5, 0.75, 1, 2, 3, 4, 6, 8, 12, 24 h), blood samples were collected in sodium heparin-containing tubes and centrifuged to separate plasma, and then frozen at −80 °C until analysis. Before analysis, plasma samples were thawed on ice. Subsequently, 900 μL of methanol with 0.1% formic acid was added to 100 μL of plasma. The mixture was vortexed for 3 min followed by centrifugation at 10,000 rpm for 10 min. Then, 800 μL of supernatant was collected and dried using flowing nitrogen gas. The residue was reconstituted in 80 μL of methanol with 0.1% formic acid, vortexed for 3 min, and centrifuged at 10,000 rpm for 10 min. DMY in the supernatant was quantitatively analyzed using HPLC. DMY concentration in the plasma at each time point was calculated, and the plasma drug concentration-time curves for each formulation were plotted. Pharmacokinetic parameters of area under the curve (AUC), mean retention time (MRT), half-life (t_1/2_), clearance (CL), apparent volume of distribution (V), maximum drug concentration time (T_max_), and maximum plasma drug concentration (C_max_) were analyzed using Data Analysis System (DAS, version 2.0, DrugChina, Beijing, China).

### 2.8. In Vivo Anti-NAFLD Efficacy

C57BL/6J mice (male, 4 w, n = 55) were used in this study. The healthy group (n = 8) were fed on a normal chow diet (NCD), while the remaining mice were given a high fat diet (HFD) to introduce the NAFLD model. After 12 weeks of HFD feeding, modeled mice with body weight gain > 60% were selected and randomly divided into the following groups: model, DMY, DMY+vehicle, DMY-hNE, and MET (n = 8 for each group). All groups of HFD feed were replaced with NCD feed. Before treatment, mice were fed in this condition for 3 days to get used to their new environment. Mice in group DMY, DMY+vehicle and DMY-hNE were i.g. administrated with DMY suspension, DMY + blank hNE suspension, and DMY-hNE at the equivalent dose of 25 mg DMY/kg. Metformin was selected as the positive control [8,35], and mice in group MET were i.g. administrated with 250 mg/kg metformin solution. Mice in group Model were i.g. treated with the same volume of water as a placebo. The body weight of mice in each group was measured every week. After 6 weeks, mice were fasted overnight, anesthetized to collect blood samples, and then sacrificed to collect liver tissues. Blood samples were centrifuged to separate plasma, and the levels of TG, TC, LDL, HDL, ALT, AST, ALP, BUN, and MDA were determined. Liver tissues were weighted, and a part of the liver tissue was frozen under −80 °C or fixed in 4% paraformaldehyde solution to prepare oil red O (ORO) staining and hematoxylin and eosin (H&E) staining sections. The frozen tissues were thawed, homogenized, centrifuged to detect ALT, AST, ALP, GGT, NO, MDA, GSH, SOD, and H_2_O_2_ levels.

### 2.9. Safety Evaluation

To evaluate the in vivo safety of DMY-hNE, twenty-five C57BL/6J mice (male, 8w) were randomly divided into five groups (n = 5): Healthy, DMY, DMY+vehicle, DMY-hNE, and MET. Mice in group DMY, DMY+vehicle and DMY-hNE were daily i.g. administrated with DMY suspension, DMY + blank hNE suspension, and DMY-hNE at the equivalent dose of 25 mg DMY/kg, while mice in group MET were i.g. administrated with 250 mg/kg metformin solution. After 6 weeks, all the mice were sacrificed, and the main organs (heart, liver, spleen, lung, kidney) and blood samples were collected. Blood samples were centrifugated to separate plasma, and AST, ALT, and BUN levels were detected. The main organs were fixed in 4% paraformaldehyde solution to prepare tissue sections and H&E staining.

### 2.10. Statistics

GraphPad Prism software (version 8.0, Dotmatics Co., Boston, MA, USA) was used to process data. The data were presented as mean ± standard error of mean (SEM). The student’s *t*-test was used to assess the differences between the groups. When *p* < 0.05, the differences were considered significant.

## 3. Results and Discussion

### 3.1. Preparation and Characterization of DMY-hNE

The application of DMY in the early stages of NAFLD is crucial in preventing its further progression to cirrhosis or liver-related diseases [16]. DMY has demonstrated a great deal of promise in the management of NAFLD [36,37]. Due to its limited bioavailability, DMY requires a higher concentration to effectively target tissue. In this study, a liver-targeted biomimetic nano-emulsion, i.e., DMY-hNE, was designed to increase in vivo absorption and hepatic effective concentration of DMY. DMY-hNE was prepared by applying the two-step emulsification method. PLA was selected to construct the DMY-loaded stiff core due to its rigid mechanical properties [38]. Lipid matrices, such as lecithin, cholesterol, and glycerol monooleate, were used to form the biomimetic soft shell of DMY-hNE.

**Figure 2:**
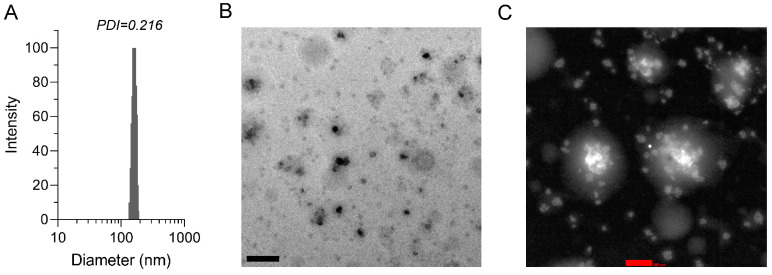
Characterization of DMY-hNE. (**A**) Size measured by DLS, (**B**) representative FTEM image and (**C**) STEM image of DMY-hNE in water. Scale bars represent 500 nm (black) and 200 nm (red).

The mean diameter of DMY-hNE was 164.6 ± 2.9 nm, while the zeta potential was −5.15 ± 0.84 mV determined by DLS zeta plus (Figure 2A). Studies have revealed that nanoparticles with a size of 50–200 nm have the properties of long-time blood circulation, tissue targeted delivery, and reduced in vivo clearance [39]. DMY-hNE has a size <200 nm, which is beneficial to intestinal drug penetration and liver accumulation. The stiff-soft (core-shell) structure of DMY-hNE was observed by FTEM and STEM. As displayed in Figure 2B, C, the soft shell of DMY-hNE exhibited low contrast, while the stiff core was in high contrast. In addition, the stiff cores distributed in the soft shell.

Encapsulation efficiency is an important characteristic to evaluate the quality of nano-formulation. DMY-hNE showed a high encapsulation efficiency of 99.4 ± 0.5%, which indicated excellent quality and low drug leakage in the preparation process.

### 3.2. Stability and In Vitro DMY Release

**Figure 3:**
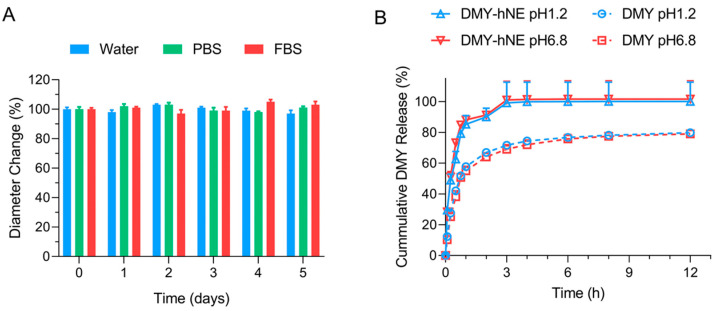
(**A**) Stability and (**B**) in vitro release profiles of DMY-hNE.

Following the successful preparation of DMY-hNE, we examined the stability of DMY-hNE in water, FBS, and PBS at 4 °C. The particle size of DMY-hNE exhibited minimal fluctuation over 5 days, suggesting good store stability of DMY-hNE (Figure 3A). We further studied the in vitro release behavior of DMY by simulating the environment of a fasting stomach and small intestine at pH 1.2 and pH 6.8, respectively. In the release media of pH 1.2 and pH 6.8, DMY and DMY-hNE showed a rapid release within the first 1 h. (Figure 3B). Compared to free DMY suspension, a faster cumulative DMY release was observed in the DMY-hNE group within 12 h, both in pH 1.2 and pH 6.8 media, which may be attributed to the increased dissolving and controlled release of small-sized DMY-hNE. These results confirmed that DMY-hNE was stable under storage and could enhance the release of DMY.

### 3.3. Stomach and Small Intestine Uptake

**Figure 4:**
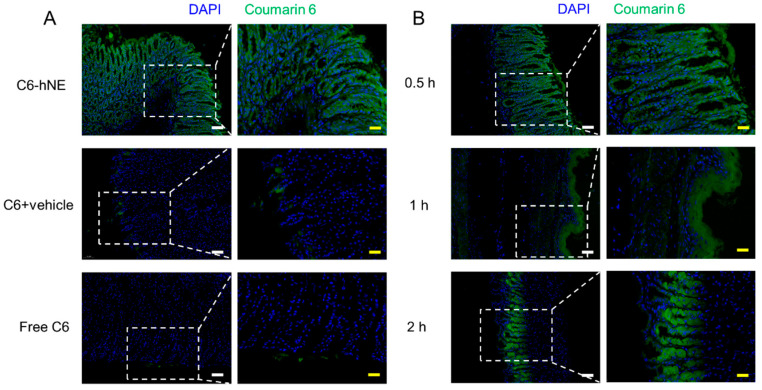
(**A**) Stomach uptake of healthy mice i.g. treated with C6-hNE, C6 + vehicle, free C6 at 0.5 h. (**B**) Stomach uptake of healthy mice i.g. treated with C6-hNE at different time intervals (0.5, 1, 2 h). Scale bars represent 50 μm (white) and 20 μm (yellow).

**Figure 5:**
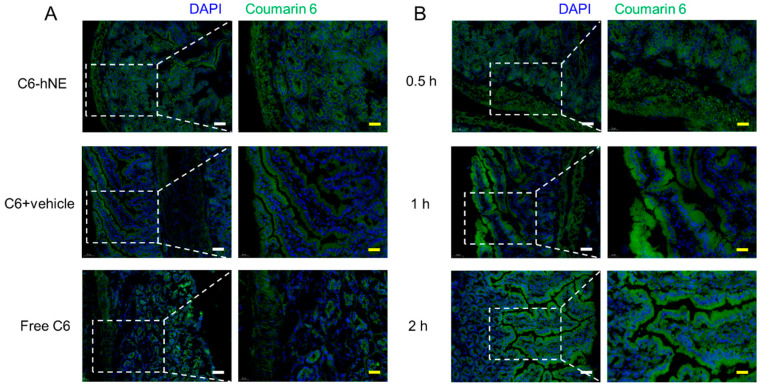
(**A**) Small intestine uptake of healthy mice i.g. treated with C6-hNE, C6 + vehicle, free C6 at 0.5 h. (**B**) Small intestine uptake of healthy mice i.g. treated with C6-hNE at different time intervals (0.5, 1, 2 h). Scale bars represent 50 μm (white) and 20 μm (yellow).

To evaluate whether the designed hNE can increase the uptake of drugs in the GI as expected, free C6, C6 + blank hNE suspension, and C6-hNE were i.g. administrated to mice, respectively. As shown in Figure 4A and Figure 5A, the uptake fluorescence intensity of C6-hNE in the stomach and small intestine was higher than that of the C6 and C6 + vehicle, indicating the promoted uptake of hNE as a smart oral nanocarrier. Moreover, C6-hNE was mainly distributed on the surface of intestinal villi at 0.5 h, and the nano-emulsion almost penetrated into the inner part of the intestinal villi at 2 h (Figure 4B and Figure 5B). These results indicated that hNE could effectively pass through the mucus layer and transport drugs from the villus of the small intestine to the basal layer in a time-dependent manner. The enhanced release of DMY-hNE facilitated sufficient uptake of DMY in the stomach and intestine. Additionally, DMY-hNE with a soft-stiff structure promoted the penetration of DMY into the mucus layer and its uptake by epithelial cells of the GI.

### 3.4. In Vivo Liver Targeted Delivery

**Figure 6:**
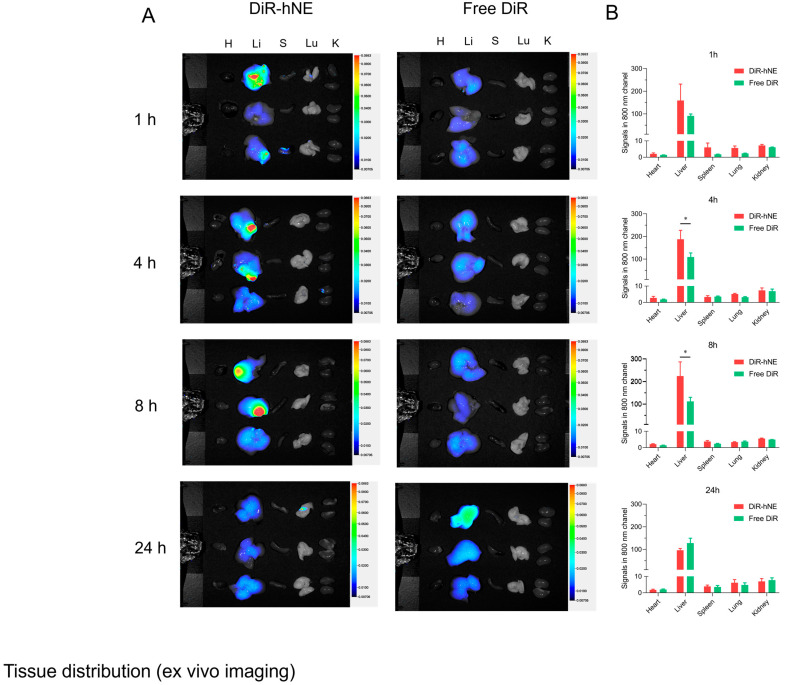
Biodistribution and liver-targeting effect of DiR-hNE. (**A**) Representative ex vivo images and (**B**) quantitative analysis results in different time intervals. * *p* < 0.05, vs. free DiR.

To confirm the liver-targeted delivery of DMY-hNE, DiR was selected as a probe to label DMY-hNE for in vivo fluorescence tracking. Mice were i.g. administrated with free DiR and DiR-hNE. At predetermined time intervals (1, 4, 8, 24 h), the fluorescence signal in the main organs (heart-H, liver-Li, spleen-S, lung-Lu, kidney-K) was analyzed by a living imaging system. As shown in Figure 6A, the fluorescence signal was largely distributed in liver tissue following i.g. administration of free DiR and DiR-hNE. Significantly, the fluorescence signal of DiR-hNE was stronger than that of free DiR. Moreover, the fluorescence signal in the liver tissue of mice in group DiR-hNE gradually increased and peaked at 8 h in a time-dependent manner. The enhanced fluorescence signals of DiR-hNE may be attributed to the proper particle size (<200 nm) which led to liver-targeted accumulation [39,40]. For further precise analysis, the fluorescence intensity was quantitatively measured. Fluorescence quantitative results showed that the accumulation of NE at 4 h and 8 h was significantly increased compared with free DiR (*p* < 0.05). Moreover, the quantitative fluorescence intensity in the liver was significantly higher than that in other organs (Figure 6B). These results demonstrated that the nanocarrier of hNE could successfully deliver the drug to target liver tissue.

### 3.5. Pharmacokinetics

**Figure 7:**
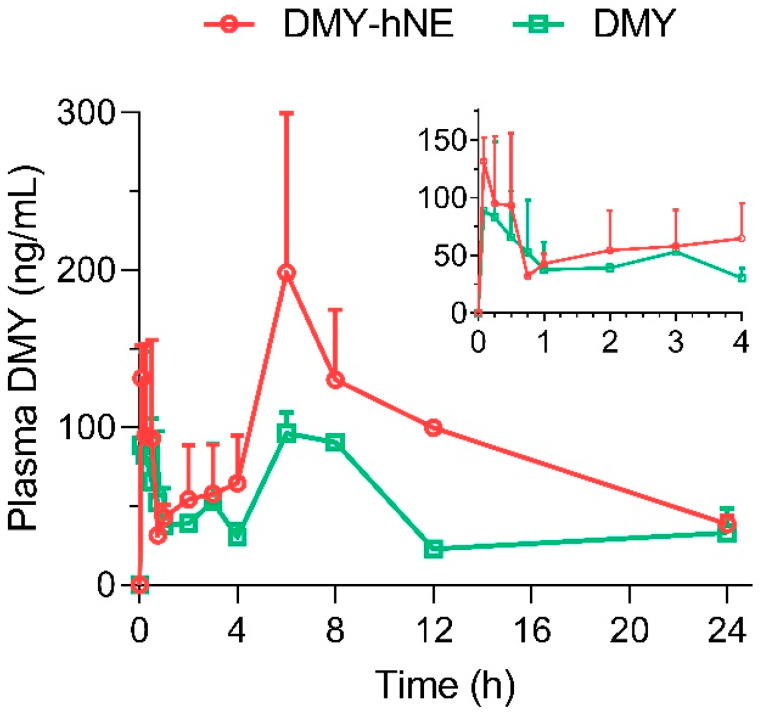
In vivo pharmacokinetic profiles of DMY-hNE. DMY-hNE and free DMY were orally administered to rats at a dose of 25 mg DMY/kg, respectively (n = 3).

The plasma concentration–time profiles and pharmacokinetic parameters of DMY suspension and DMY-hNE following i.g. administration are presented in Figure 7 and Table 1. As the results indicated, DMY suspension and DMY-hNE both reached their first peak at 0.25 h, followed by their second peak at 6 h. This double peak phenomenon may be associated with the multiple sites of absorption in the GI. Meanwhile, the distribution of DMY in the liver showed a time-dependent pattern and peaked around 8 h, which may allow the drugs to reach a second absorption in the intestine at 6 h. The C_max_ of DMY-hNE was 215.66 ± 76.47 μg L^−1^, which was 1.8 times that of DMY. In addition, CL of DMY-hNE was significantly lower than that of DMY. At the same dose, AUC of DMY-hNE increased significantly (*p* < 0.01) to ~2.0 times that of free DMY suspension. In general, DMY-hNE exhibited prolonged in vivo accumulation and lower elimination compared to DMY suspension, suggesting the increased oral bioavailability of DMY via the nanocarrier. The bioavailability and pharmacokinetic profile of DMY could be affected by oxidative degradation, gastrointestinal instability, metabolic transformation, and poor permeability [19]. In our study, the increased bioavailability of DMY in DMY-hNE may be attributed to increased dissolving/faster release in the gastrointestinal tract. In addition, DMY-hNE is a lipid-based delivery system with a soft shell, which was helpful for penetrating the mucus layer [41].

### 3.6. In Vivo Anti-NAFLD Efficacy of DMY-hNE

**Figure 8:**
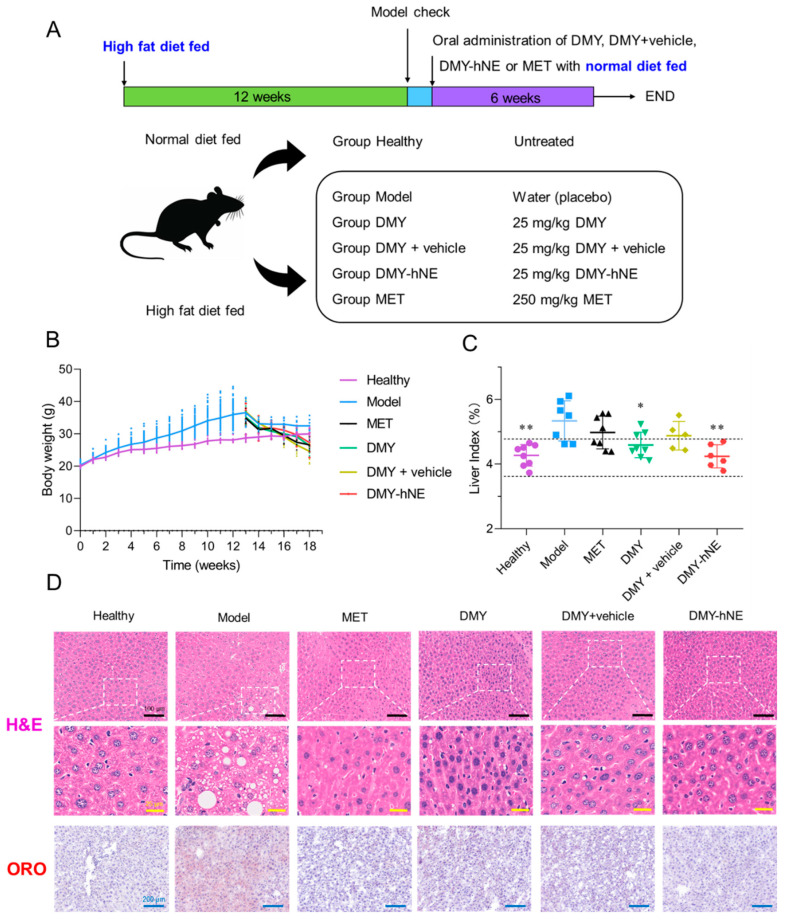
In vivo anti-NAFLD efficacy of DMY-hNE. Free DMY, DMY + vehicle, and DMY-hNE were orally administrated to NAFLD mice at a dose of 25 mg DMY/kg, respectively, while MET was orally administrated at a dose of 250 mg MET/kg. (**A**) Schematic illustration of the experiment, (**B**) body weight changes, (**C**) liver index (Black dashed lines represent liver index range of healthy mice), and (**D**) representative HE and oil red O staining of healthy mice and NAFLD mice treated with water (placebo), free DMY, DMY + vehicle, DMY-hNE and metformin for 6 weeks. Scale bars represent 100 μm (black), 25 μm (yellow) and 200 μm (blue). * *p* < 0.05, ** *p* < 0.01, vs. group Model.

Considering that DMY-hNE improved the oral bioavailability of DMY, we further evaluated the in vivo anti-NAFLD efficacy of DMY-hNE. The NAFLD mouse model was established by feeding C57BL/6J mice with HFD for 12 weeks, according to the literature [42,43]. As observed in Figure 8B, mice in group Model maintained on an HFD produced a progressive elevation in body weight compared to the healthy mice. Obesity is a major driver in the development of NAFLD, while a healthy lifestyle and weight loss are effective in the prevention and treatment of NAFLD [5]. Hence, after the introduction of HFD and the typical obesity of NAFLD appeared, all HFD were switched to NCD and appropriate medication or a placebo were administrated to the mice, where this kind of NAFLD model (i.e., early NAFLD) was considered self-healing and close to clinical conditions. The body weight of NAFLD mice decreased after the HFD was replaced by NCD, and mice treated with DMY suspension, DMY + blank hNE suspension, DMY-hNE, and metformin decreased more obviously compared to those in group Model. The liver index in group DMY-hNE was also lower than that in group Model, which was similar to healthy mice (Figure 8C). These results indicated that DMY-hNE ameliorated HFD-induced obesity. H&E staining was used to study histological changes of liver in mice. As presented in Figure 8D, liver sections of group Model showed typical cytoplasmic fat vacuoles. Macrovesicular steatosis was less severe in mice treated with DMY-hNE than those treated with DMY suspension. Similar findings were also verified by morphological analysis of liver sections stained with oil red O. Liver sections of group Model showed steatosis and many red-stained lipid droplets in the cytoplasm. In addition, the number of lipid droplets significantly reduced in group DMY-hNE, which was better than group DMY. These results showed that DMY-hNE had the effect of reducing lipid accumulation in NAFLD mice and was superior to free DMY, which might be attributed to the enhanced digestive absorption and improved in vivo pharmacokinetic behavior of DMY-hNE.

### 3.7. Biochemistry Analysis

**Figure 9:**
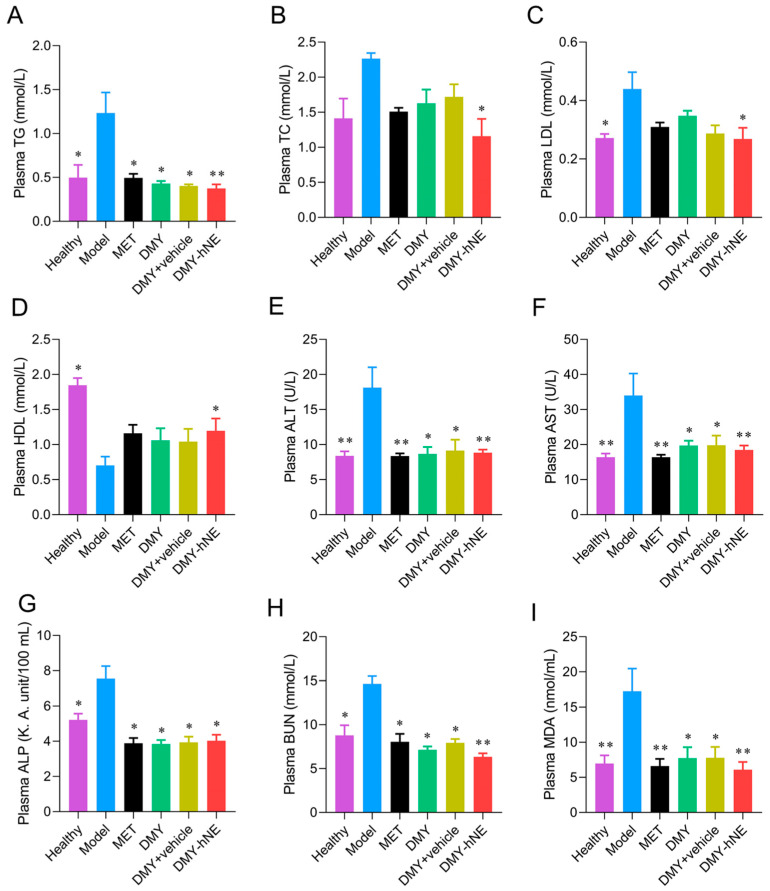
Blood biochemistry. Plasma (**A**) TG, (**B**) TC, (**C**) LDL, (**D**) HDL, (**E**) ALT, (**F**) AST, (**G**) ALP, (**H**) BUN, and (**I**) MDA levels of healthy mice and NAFLD mice treated with water (placebo), free DMY, DMY + vehicle, DMY-hNE and metformin for 6 weeks. * *p* < 0.05, ** *p* < 0.01, vs. group Model.

In order to further investigate the possible mechanism of the promoted anti-NAFLD efficacy, blood biochemistry and liver tissue biochemistry experiments were conducted. Briefly, blood biochemistry was assessed. Mice in group Model exhibited lower plasma HDL levels and higher plasma TG, TC, and LDL levels, as well as higher plasma AKP, ALT, and AST levels compared to healthy mice (Figure 9A–G). It was suggested that the early NAFLD animal model induced by a high-fat diet was successful. Moreover, mice treated with DMY and DMY-hNE had lower levels of plasma AKP, ALT, and AST, higher levels of HDL, and reduced accumulation of TG, TC, and LDL when compared with the mice in group Model (Figure 9A–G). These results indicated that the intervention of DMY-hNE ameliorated NAFLD-related liver injury and lipid accumulation. In addition, TC in group DMY+vehicle was slightly higher than that in group DMY, which might be related to the existence of cholesterol in the blank vehicle. To further study the metabolism and inflammation status of early NAFLD mice treated with various formulations, plasma MDA was analyzed. The results (Figure 9I) revealed that MDA levels were elevated in NAFLD model mice without treatment, which were in contrast ameliorated by DMY intervention. Group DMY-hNE exhibited better MDA amelioration than group DMY and group DMY + vehicle, which was in accordance with the results in Section 3.6.

**Figure 10:**
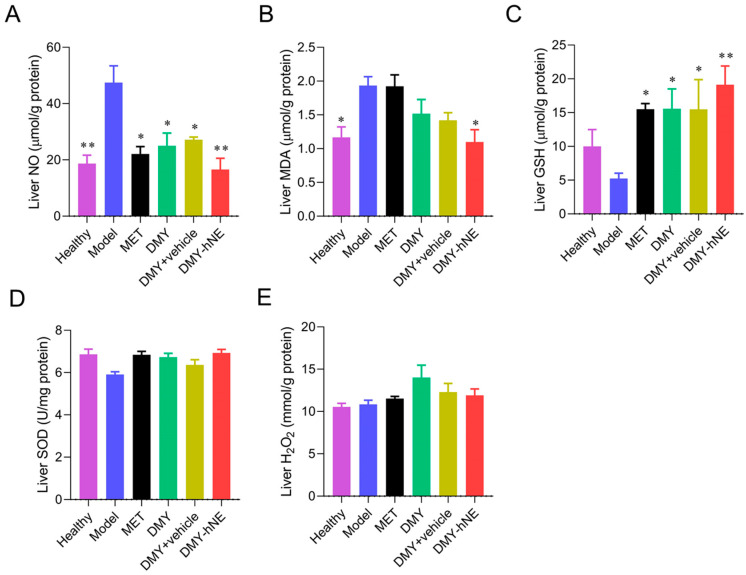
Liver tissue biochemistry. Liver (**A**) NO, (**B**) MDA, (**C**) GSH, (**D**) SOD, and (**E**) H_2_O_2_ levels of healthy mice and NAFLD mice treated with water (placebo), free DMY, DMY + vehicle, DMY-hNE and metformin for 6 weeks. * *p* < 0.05, ** *p* < 0.01, vs. group Model.

Liver tissue biochemistry was further studied. As shown in Figure 10A–E, liver tissue NO and MDA levels were elevated in group Model compared to group Healthy, and this elevation was ameliorated by the intervention of DMY-hNE, DMY + vehicle, and free DMY. These findings suggested that DMY-hNE ameliorated inflammation in liver tissue. To further investigate the mechanism of the protective effect of DMY-hNE against early NAFLD, liver tissue oxidative stress indexes were measured. Following HFD fed, GSH and SOD levels in liver tissue were decreased. GSH levels were up-regulated with drug intervention, while SOD levels did not change significantly. Slight elevation of liver tissue H_2_O_2_ was observed after dietary induction of HFD. The insignificant changes of SOD and H_2_O_2_ levels indicated that the hepaprotective effect of DMY may be not through adjusting oxidative stress, but through the inflammation pathway.

### 3.8. Safety Evaluation

**Figure 11:**
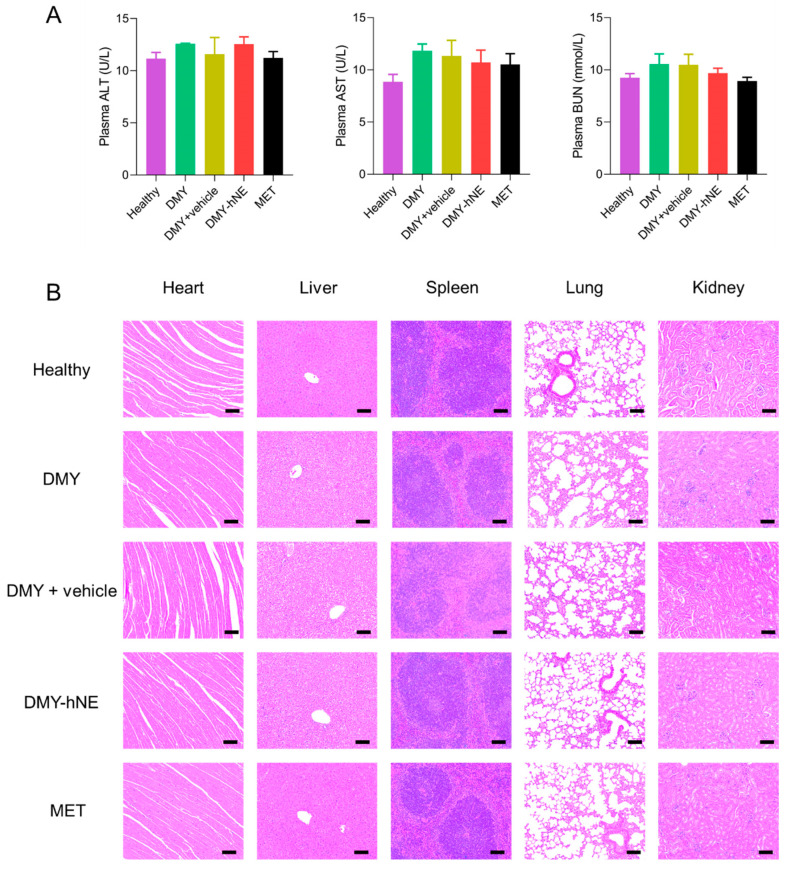
In vivo safety evaluation of DMY-hNE. (**A**) Plasma ALT, AST, BUN levels and (**B**) representative HE staining of the main organs of healthy mice treated with water, free DMY, DMY + vehicle, DMY-hNE and metformin for 6 weeks. Scale bars represent 100 μm.

To evaluate the in vivo safety of DMY-hNE, C57BL/6 mice were i.g. administrated with the same interventions as the in vivo anti-NAFLD experiment in Section 3.6. During i.g. administration, no death or abnormal behavior of mice was observed. The plasma AST, ALT, and BUN levels in group DMY, DMY + vehicle, DMY-hNE, and MET were all within the normal range (Figure 11A). Moreover, H&E staining results of the main organs (heart, liver, spleen, lung and kidney) showed no pathological changes (Figure 11B), which demonstrated the safety of DMY-hNE via oral administration.

## 4. Conclusions

In conclusion, our study successfully developed a novel structure of stiff-soft hybrid biomimetic nano-emulsion, which significantly promoted uptake in the stomach and small intestine, increased drug accumulation in the liver, and enhanced the oral bioavailability of DMY. Moreover, in the early NAFLD mice model, DMY-hNE enhanced the therapeutic efficacy of DMY by inhibiting lipid accumulation and decreasing tissue inflammation, which implied that DMY-hNE was a promising platform for liver-targeted delivery and treatment of NAFLD. In addition, the ingredients of hNE are all pharmaceutical adjuvants and approved by the FDA, which provides the potential to fabricate it in the industry on a large scale.

## Figures and Tables

**Table 1:** Summarized pharmacokinetic parameters of DMY after oral administration of DMY-hNE and free DMY to rats at a dose of 25 mg DMY/kg, respectively (n = 3).

Parameters	Unit	DMY-hNE	DMY
AUC ^[A]^	μg L^−1^ h	2121.80 ± 74.20 ^[^**^]^	1065.70 ± 90.34
MRT_0-t_^[B]^	h	10.08 ± 0.56 ^[ns]^	10.74 ± 0.23
t_1/2_^[C]^	h	8.71 ± 3.47 ^[ns]^	11.71 ± 4.55
CL ^[D]^	L h^−1^ kg^−1^	7.66 ± 0.63 ^[ns]^	12.65 ± 4.28
V ^[E]^	L kg^−1^	94.75 ± 30.51 ^[^*^]^	199.76 ± 10.85
C_max_^[F]^	μg L^−1^	215.66 ± 76.47 ^[ns]^	117.34 ± 16.76
T_max 1_^[G]^	h	0.25	0.25
T_max 2_	h	6	6

Note: ^[A]^ Area under the curve, ^[B]^ mean retention time, ^[C]^ half-life, ^[D]^ clearance, ^[E]^ apparent volume of distribution, ^[F]^ maximum drug concentration, ^[G]^ maximum drug concentration time. ^[^*^]^ *p* < 0.05, vs. DMY; ^[^**^]^ *p* < 0.01, vs. DMY; ^[ns]^ no significant difference, *p* > 0.05, vs. DMY.

## Data Availability

Data are available from the corresponding author on reasonable request.
